# HIF-1α Dependent Upregulation of ZIP8, ZIP14, and TRPA1 Modify Intracellular Zn^2+^ Accumulation in Inflammatory Synoviocytes

**DOI:** 10.3390/ijms22126349

**Published:** 2021-06-14

**Authors:** Noriyuki Hatano, Masaki Matsubara, Hiroka Suzuki, Yukiko Muraki, Katsuhiko Muraki

**Affiliations:** Laboratory of Cellular Pharmacology, School of Pharmacy, Aichi-Gakuin University, 1-100 Kusumoto, Chikusa, Nagoya 464-8650, Japan; nhatano@dpc.agu.ac.jp (N.H.); acetyl.saritirusan@gmail.com (M.M.); hsuzuki@dpc.agu.ac.jp (H.S.); ymuraki@helen.ocn.ne.jp (Y.M.)

**Keywords:** TRPA1, ZIP8, ZIP14, intracellular Zn^2+^ concentration, synoviocytes, inflammation, hypoxia-induced factor 1α (HIF-1α)

## Abstract

Intracellular free zinc ([Zn^2+^]_i_) is mobilized in neuronal and non-neuronal cells under physiological and/or pathophysiological conditions; therefore, [Zn^2+^]_i_ is a component of cellular signal transduction in biological systems. Although several transporters and ion channels that carry Zn^2+^ have been identified, proteins that are involved in Zn^2+^ supply into cells and their expression are poorly understood, particularly under inflammatory conditions. Here, we show that the expression of Zn^2+^ transporters ZIP8 and ZIP14 is increased via the activation of hypoxia-induced factor 1α (HIF-1α) in inflammation, leading to [Zn^2+^]_i_ accumulation, which intrinsically activates transient receptor potential ankyrin 1 (TRPA1) channel and elevates basal [Zn^2+^]_i_. In human fibroblast-like synoviocytes (FLSs), treatment with inflammatory mediators, such as tumor necrosis factor-α (TNF-α) and interleukin-1α (IL-1α), evoked TRPA1-dependent intrinsic Ca^2+^ oscillations. Assays with fluorescent Zn^2+^ indicators revealed that the basal [Zn^2+^]_i_ concentration was significantly higher in TRPA1-expressing HEK cells and inflammatory FLSs. Moreover, TRPA1 activation induced an elevation of [Zn^2+^]_i_ level in the presence of 1 μM Zn^2+^ in inflammatory FLSs. Among the 17 out of 24 known Zn^2+^ transporters, FLSs that were treated with TNF-α and IL-1α exhibited a higher expression of *ZIP8* and *ZIP14*. Their expression levels were augmented by transfection with an active component of nuclear factor-κB P65 and HIF-1α expression vectors, and they could be abolished by pretreatment with the HIF-1α inhibitor echinomycin (Echi). The functional expression of ZIP8 and ZIP14 in HEK cells significantly increased the basal [Zn^2+^]_i_ level. Taken together, Zn^2+^ carrier proteins, TRPA1, ZIP8, and ZIP14, induced under HIF-1α mediated inflammation can synergistically change [Zn^2+^]_i_ in inflammatory FLSs.

## 1. Introduction

Zinc is an essential dietary metal that mainly functions as a cofactor for carbohydrates, lipids, nucleic acids, and proteins. In particular, zinc plays an obligatory role in immune function, the regulation of C-C chemokine production [1], and activation of mast cells via FcεRIs [2,3]. Other roles of this metal, in the form of the so-called free zinc (Zn^2+^), as a component of cellular signal transduction, lipopolysaccharide-induced signal transduction in monocytes [4], and cytoprotection of lung epithelia in inflammation [5], have been suggested. In addition, the importance of Zn^2+^ in neuronal cells and its involvement in neuronal death is well-documented [6,7]. Therefore, zinc homeostasis is a major focus of research in biology, and zinc transporters, such as ZnT (ZnT1- ZnT10) and ZIP (ZIP1-ZIP14), respectively, functioning as Zn^2+^ exporters and importers, have been identified as regulators of this metal ion in a physiological milieu. However, the molecular mechanisms that underlie the regulation of ZnT and ZIP expression remain unclear. Although transcriptional and non-transcriptional regulation of the expression of some zinc transporters have been revealed [5,8,9,10,11,12], the present understanding of this regulatory phenomenon is rather limited. Furthermore, the control of Zn^2+^ is complex and multiple mechanisms are involved: zinc transporters that are located at intracellular organelles, protein binding, and metallothioneins in a redox sensitive pool of zinc. Therefore, cellular Zn^2+^ represents a small proportion of the total zinc pool.

TRPA1 is widely expressed in neuronal and non-neuronal cells, and it acts as a biological sensor that is activated by environmental irritants and oxidative- and thiol-reactive compounds [13,14,15,16,17]. Because transgenic mice lacking TRPA1 exhibit suppressed sensitivity to mechanical stimulation, cold stimuli, and TNF-α-induced mechanical hyperalgesia [18,19,20,21,22], TRPA1 has been identified as a nociceptor mediating acute and inflammatory pain [21,22,23,24,25]. On the other hand, a previous study from our group revealed the transcriptional induction of TRPA1 by inflammation via HIF-1α as a mechanism controlling inflammatory cytokine release [26]. A unique property of TRPA1 is that the channel can be activated by a nanomolar concentration range of [Zn^2+^]_i_ and imports extracellular Zn^2+^ ([Zn^2+^]_o_), which indicates that TRPA1 is a potential Zn^2+^ transporter regulating zinc homeostasis [27,28]. Indeed, endogenous [Zn^2+^]_i_ intrinsically activates TRPA1 in inflammatory FLSs [26]. However, whether the [Zn^2+^]_i_ level in inflammatory cells is high enough to activate TRPA1 remains debatable, even though [Zn^2+^]_i_ is known to be highly potent against TRPA1 [27,29].

In this study, we show that the expression of Zn^2+^ transporters ZIP8 and ZIP14 is increased via nuclear factor-κB (NF-κB) signaling and the downstream activation of the transcription factor HIF-1α in FLSs pretreated with TNF-α and IL-1α. The resultant intracellular Zn^2+^ accumulation could activate TRPA1, leading to increased basal [Zn^2+^]_i_ and evoking Ca^2+^ oscillations. Assays with fluorescent Zn^2+^ indicators revealed that the basal [Zn^2+^]_i_ concentration was significantly higher in TRPA1-expressing HEK cells and inflammatory FLSs. Therefore, inflammatory conditions induce the expression of Zn^2+^ carrier proteins TRPA1, ZIP8, and ZIP14, which, in turn, synergistically change [Zn^2+^]_i_ in inflammatory FLSs.

## 2. Results

### 2.1. Zn^2+^-Dependent TRPA1 Activation and [Zn^2+^]_i_ Components in TRPA1-Expressing Cells

**Figure 1:**
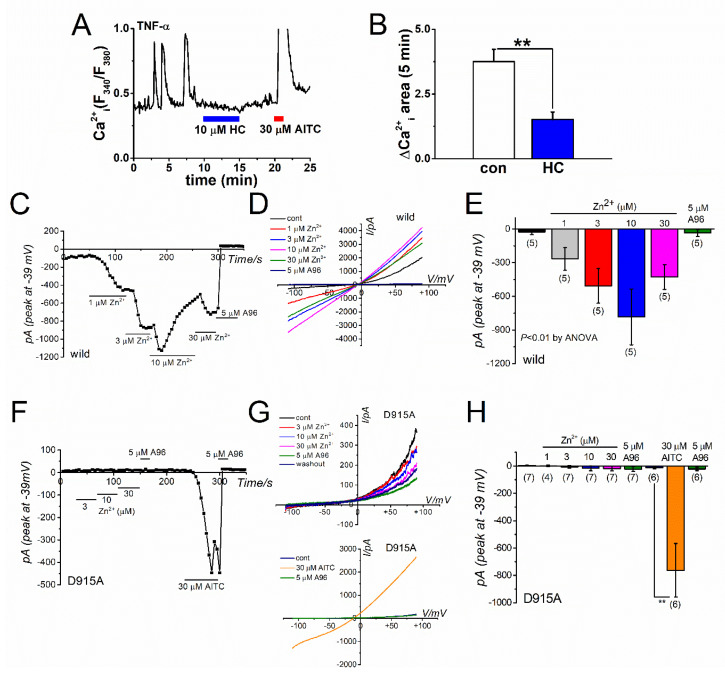
Zn^2+^-dependent TRPA1 activation. (**A**) Intrinsic Ca^2+^ oscillations sensitive to the TRPA1 antagonist HC observed on treating inflammatory FLSs with 10 U TNF-α for 24 h. Presence of the TRPA1 agonist AITC (30 μM) evoked a larger Ca^2+^ response, which was scaled out. (**B**) Inhibitory effects of 10 μM HC on Ca^2+^ oscillations in inflammatory FLSs. Mean data of ΔCa^2+^_i_ response area in a 5-min window with and without HC are pooled (*n* = 13; three independent experiments; ** *p* < 0.01). Comparison of Zn^2+^-induced TRPA1 channel currents in (**C**–**E**) wild TRPA1 (wild; five independent experiments; ** *p* < 0.01 by ANOVA) and (**F**–**H**) a mutant TRPA1 (D915A; 4–7 independent experiments; ** *p* < 0.01 by Tukey-Kramer test) in the channel pore. The mutant TRPA1 was generated by replacing an aspartic acid residue at position 915 of the wild TRPA1 with an alanine residue. Cells expressing the mutant channel were superfused with SBS and dialyzed with Cs-aspartate rich pipette solution including 0.3 μM Ca^2+^. Ramp waveform voltage pulses from −110 to +90 mV were applied for 100 ms every 5 s at a holding potential of −10 mV. Each cell was exposed to Zn^2+^ to confirm its effects on membrane currents at −39 mV, whereas the contamination by Cl^-^ currents was negligible. After treatment with the final concentration of Zn^2+^, 5 μM A-967079 (A96) was applied to assess the TRPA1 channel components giving rise to conductance. To confirm functional expression of the mutant TRPA1, AITC (30 μM) and A96 (5 μM) were used. A representative time-course change and current-voltage relationship of Zn^2+^- or AITC-induced TRPA1 currents in a HEK cell with (**C**,**D**) wild and (**F**,**G**) D915A channels. The peak values of inward current induced by (**E**) Zn^2+^- or (**F**) AITC at −39 mV were averaged and plotted.

In a previous study, we have shown that TNF-α and IL-1α induce *TRPA1* gene expression via NF-κB signaling and the downstream activation of HIF-1α in FLSs. Indeed, 50% FLSs that were treated with 10 U TNF-α for 24 h exhibited intrinsic Ca^2+^ oscillations, which were inhibited by the TRPA1 antagonist HC-030031 (HC) (Figure 1A shows a representative cell response and Figure 1B plots the overall change; see also [26]). These TRPA1-dependent Ca^2+^ oscillations were effectively inhibited by a membrane-permeable Zn^2+^ chelator N,N,N’,N-tetrakis(2-pyridylmethyl)ethylenediamine (TPEN, [26]), indicating that [Zn^2+^]_i_ acts as an endogenous TRPA1 agonist and activates the induced TRPA1. Consistent with these observations, a cumulative application of Zn^2+^ in the concentration range of 1 to 30 μM activated inward currents in TRPA1 expressing HEK (HEK-TRPA1, wild) cells, which were abolished by the selective TRPA1 antagonist A-967079 (A96, Figure 1C–E). In contrast, Zn^2+^ did not induce any inward currents in HEK cells that were transfected with a mutant TRPA1 (D915A, Figure 1F–H), in which the channel-pore is rigid against Zn^2+^, which suggests that active TRPA1 can supply Zn^2+^ into cells [27,29]. Not being directly evident, these results may suggest that intracellular Zn^2+^ can activate TRPA1-dependent Ca^2+^ and Na^+^ influx through TRPA1.

**Figure 2:**
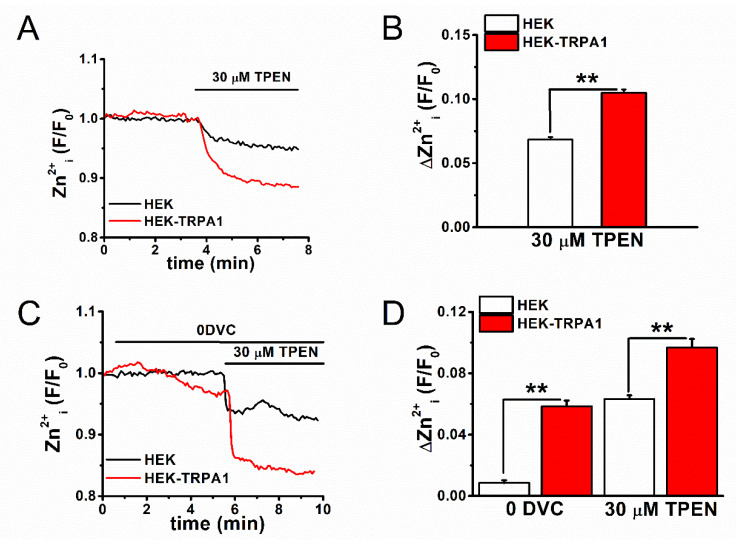
[Zn^2+^]_i_ components in HEK-TRPA1 cells. (**A**) Comparative analysis of TPEN-sensitive basal [Zn^2+^]_i_ components in control HEK and HEK-TRPA1 cells. [Zn^2+^]_i_ levels in SBS was monitored using FluoZin-3 in the presence of 30 μM TPEN. (**B**) Peak changes in [Zn^2+^]_i_ in the presence of TPEN (mean±SEM; *n* = 184 and *n* = 293, four and six independent experiments for control HEK and HEK-TRPA1 cells, respectively; ** *p* < 0.01). (**C**) Comparison of extracellular divalent cations and TPEN-sensitive basal [Zn^2+^]_i_ components assayed with FluoZin-3 in control HEK and HEK-TRPA1 cells. The extracellular divalent cations were removed without EGTA (0DVC), followed by exposure to 30 μM TPEN. (**D**) Peak changes in [Zn^2+^]_i_ in the presence of 0DVC and TPEN (mean ± SEM; *n* = 76 and *n* = 78 for HEK and HEK-TRPA1 cells, respectively; two independent experiments each, ** *p* < 0.01).

**Figure 3:**
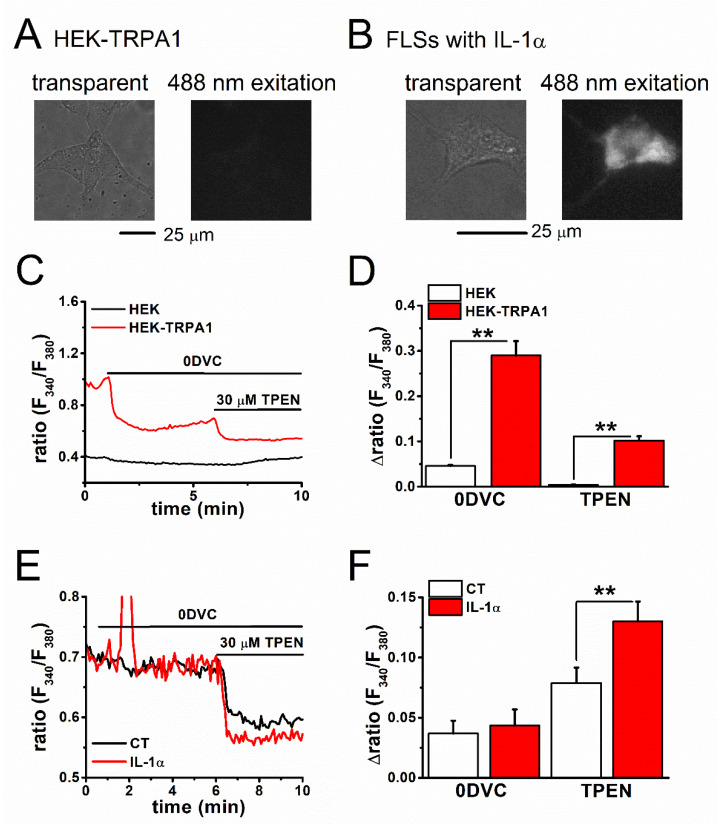
The assay of [Zn^2+^]_i_ in TRPA1-expressing cells using a Fura-2 based assay. Auto-fluorescence images of (**A**) HEK-TRPA1 cells and (**B**) FLSs with IL-1α in the absence of fluorescence indicators. Fluorescence images acquired in cells excited with light of 488 nm wavelength (right panels) and light-transmitting images (left panels) are shown. Comparison of TPEN-sensitive [Zn^2+^]_i_ components that were assayed with Fura-2 between (**C**,**D**) HEK cells with and without expression of TRPA1 or (**E**,**F**) FLSs with and without IL-1α. Cells were treated with 30 μM TPEN in the presence of 0DVC without EGTA. The fluorescence signal measured as a ratio (F_340_/F_380_) and the corresponding peak change in the presence of (**D**) 0DVC and (**F**) TPEN are plotted (mean ± SEM; *n* = 101 and *n* = 111 for HEK and HEK-TRPA1, respectively, and four independent experiments each; *n* = 33 and *n* = 31 for CT and 100 U IL-1α, respectively, and four independent experiments each; ** *p* < 0.01).

We assayed [Zn^2+^]_i_ with the fluorescent Zn^2+^ indicators FluoZin-3 and Fura-2, the zinc chelator TPEN, and the Zn^2+^ ionophore zinc-pyrithione (Pyr) to further examine the contribution of TRPA1 as a Zn^2+^ carrier in maintaining [Zn^2+^]_i_ level and identify other regulators of [Zn^2+^]_i_ in inflammatory FLSs. First, TPEN-sensitive [Zn^2+^]_i_ components were assayed using FluoZin-3 (K_d-Zn2+_: ~15 nM), which functions as a selective Zn^2+^ indicator, in cells with and without TRPA1 expression. When the TPEN-sensitive [Zn^2+^]_i_ components were compared between the control HEK (HEK) and HEK-TRPA1 cells in standard bathing solution (SBS), the [Zn^2+^]_i_ components were significantly higher in HEK-TRPA1 cells (Figure 2A,B). The control HEK and HEK-TRPA1 cells were treated with 30 μM TPEN after the removal of extracellular divalent cations without EGTA (zero divalent cation; 0DVC), as shown in Figure 2C,D. This induced substantially larger reductions in the [Zn^2+^]_i_ components in HEK-TRPA1 cells (Figure 2D), suggesting that active rapid Zn^2+^ influx through TRPA1 (0DVC-dependent) and subsequent basal [Zn^2+^]_i_ accumulation (TPEN-dependent) were prominent in HEK-TRPA1 cells. In contrast, a quantitative assay of [Zn^2+^]_i_ components in FLSs with FluoZin-3 offered limited insights, due to the large auto-fluorescence signals of these cells (Figure 3A,B). In particular, this higher background fluorescence in FLSs interacts with the FluoZin-3 signal, affecting the accurate evaluation of [Zn^2+^]_i_. When alternatively assayed with Fura-2 (K_d-Zn2+_: ~2 nM), which acts as a Ca^2+^ and Zn^2+^ indicator, 0DVC-sensitive components were again found to be higher in HEK-TRPA1 cells when compared to that in the control HEK cells, while no appreciable difference was observed between FLSs with and without IL-1α (Figure 3C–F). However, the TPEN-sensitive [Zn^2+^]_i_ components in 0DVC were significantly larger in both HEK-TRPA1 cells (Figure 3C,D) and FLSs with IL-1α (Figure 3E,F). Therefore, although Ca^2+^-dependent Fura-2 signals could mask Zn^2+^-dependent Fura-2 signals under perfusion with SBS, TPEN in the presence of 0DVC is a useful marker for evaluating Zn^2+^-dependent Fura-2 signals.

### 2.2. Estimation of Basal [Zn^2+^]_i_ Concentration in TRPA1-Expressing Cells

**Figure 4:**
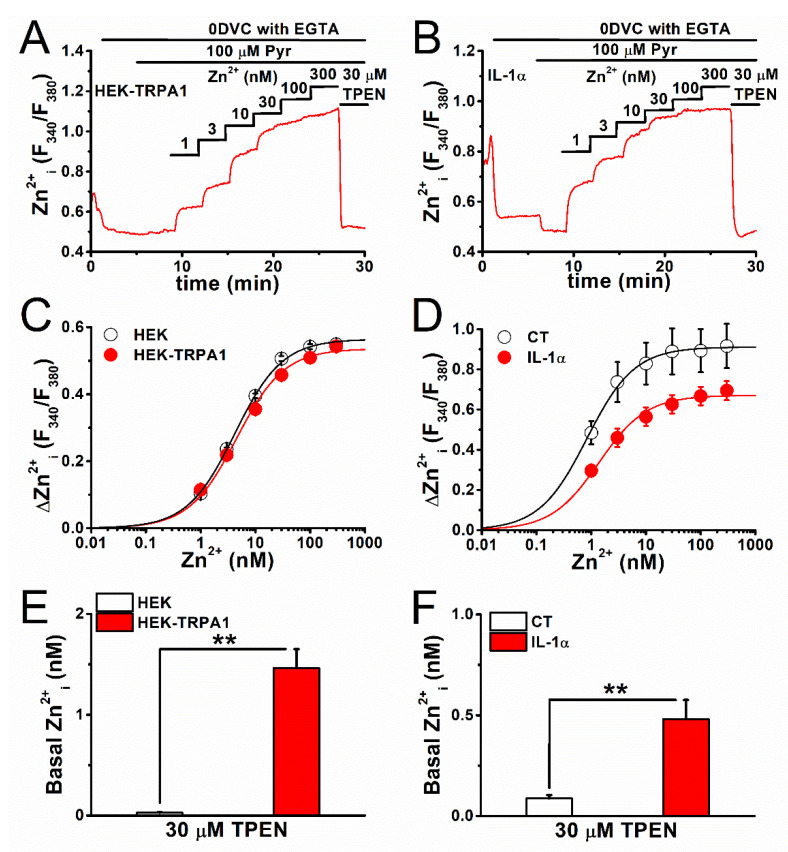
The identification of basal [Zn^2+^]_i_ concentration in TRPA1-expressing cells. Relationship between [Zn^2+^]_i_-dependent fluorescence ratio (Zn^2+^_i_ (F_340_/F_380_)) and [Zn^2+^]_i_ concentration was assayed with Fura-2 in (**A**,**C**) HEK cells and (**B**,**D**) FLSs. In the presence of 0DVC, Zn^2+^ buffered with 1 mM EGTA was cumulatively applied to (**A**) HEK-TRPA1 cells and (**B**) FLSs with 100 U IL-1α for 24 h, after permeabilization with the Zn^2+^ ionophore Pyr (100 μM). (**C**,**D**) [Zn^2+^]_i_-dependent change in fluorescence ratio (ΔZn^2+^_i_ (F_340_/F_380_)) is plotted (*n* = 94 and *n* = 89 for HEK and HEK-TRPA1 cells, respectively, and two independent experiments for each; *n* = 22 and *n* = 30 for CT and 100 U IL-1α, respectively, and three independent experiments for each). The generated dataset was fitted to a dissociation curve of Fura-2 vs. Zn^2+^ concentration (see Experimental procedures). (**E**,**F**) Data in Figure 3 and the dissociation curves (**C**,**D**) were utilized to estimate the basal [Zn^2+^]_i_ concentration in each condition. The pooled data are averaged and represented as bar charts (mean ± SEM; *n* = 101 and *n* = 111 for HEK and HEK-TRPA1 cells, respectively, and four independent experiments each; *n* = 33 and *n* = 31 for CT and 100 U IL-1α, respectively, and four independent experiments each; ** *p* < 0.01).

We constructed a dissociation curve of fluorescent signals from Fura-2 (reflecting cellular Zn^2+^ content) against [Zn^2+^]_i_ concentration to determine the [Zn^2+^]_i_ concentration in TRPA1-expressing cells (Figure 4A–H). In 0DVC buffered with EGTA (1 mM), cumulative treatment with Zn^2+^ at concentrations ranging from 1 to 300 nM was performed on HEK-TRPA1 cells (Figure 4A) and FLSs with IL-1α (Figure 4B), after the permeabilization of these cells with the Zn^2+^ ionophore Pyr (100 μM). We added 30 μM TPEN at the end of each experiment to verify the changes in Zn^2+^-dependent Fura-2 signals. The data set obtained this way was fitted to a dissociation curve of Fura-2 vs. Zn^2+^ concentration (see the Experimental Procedure) and K_d_ was calculated to be 4.2. 4.5, 0.9, and 1.5 nM for HEK cells, HEK-TRPA1 cells, FLSs without IL-1α (CT), and FLSs with IL-1α, respectively (Figure 4C,D). It is obvious that TRPA1-expressing cells had higher basal [Zn^2+^]_i_ concentration (see Methods in detail, 1.46 ± 0.19 nM in HEK-TRPA1 cells vs. 0.03 ± 0.01 nM in HEK cells, *p* < 0.01, Figure 4E; 0.48 ± 0.10 nM in FLSs with IL-1α vs. 0.09 ± 0.01 nM in CT, *p* < 0.01, Figure 4F), which suggested that TRPA1 is one of the factors to change the basal [Zn^2+^]_i_ level in TRPA1-expressing cells.

### 2.3. Mechanism of Elevation of Basal [Zn^2+^]_i_ Concentration in Inflammatory FLSs

**Figure 5:**
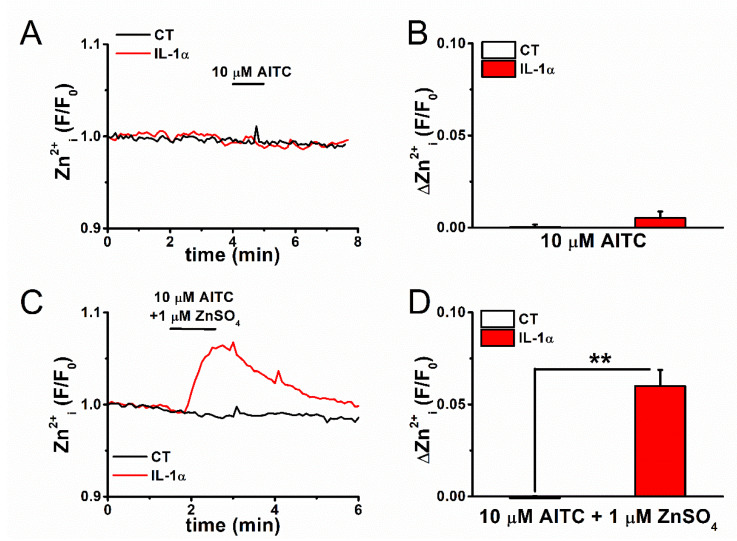
A change in [Zn^2+^]_i_ by activation of TRPA1 in FLSs. FLSs pretreated with and without 100 U IL-1α for 24 h were exposed to AITC at 10 μM, followed by monitoring of (**A**) [Zn^2+^]_i_ assayed by FluoZin-3 and (**B**) change in [Zn^2+^]_i_ (mean ± SEM; *n* = 16 and *n* = 28 for CT and IL-1α, respectively, and three independent experiments each). (**C**,**D**) These experiments were repeated in FLSs the presence of 1 μM ZnSO_4_ in the SBS to increase the driving force of Zn^2+^ (mean ± SEM; *n* = 38 and *n* = 26 for CT and 100 U IL-1α, respectively, and two independent experiments each; ** *p* < 0.01).

Although the large auto-fluorescence of FLSs complicates the use of FluoZin-3, we nevertheless attempted to detect Zn^2+^-dependent FluoZin-3 signals in FLSs. Exposure to 10 μM AITC did not induce any change in [Zn^2+^]_i_ within this short time scale in FLSs stained with FluoZin-3, even though TRPA1 was induced by pretreatment with IL-1α, as shown in Figure 5A,B. In contrast, extracellular free Zn^2+^ ([Zn^2+^]_e_) itself could permeate the TRPA1 pore (see Figure 1C–H), which suggested that [Zn^2+^]_i_ change is not detectable in FLSs within a short amount of time, even when the corresponding TRPA1 channels are activated by AITC, presumably due to the low expression of TRPA1. In fact, in the presence of 1 μM Zn^2+^ in SBS, a small but substantial Zn^2+^ response was elicited by the treatment of inflammatory FLSs with 10 μM AITC (Figure 5C,D). Therefore, TRPA1 in inflammatory FLSs functions as a Zn^2+^ carrier into cells (Figure 4), while additional Zn^2+^ carriers and other factors play obligatory roles in the regulation of cellular Zn^2+^. It is to be noted here that the [Zn^2+^]_e_ concentration in SBS used under the present experimental conditions was less than 153 nM (Supplementary Figure S1).

### 2.4. Expression and Inflammation-Mediated Transcriptional Regulation of Zn^2+^ Transporters in FLSs

**Figure 6:**
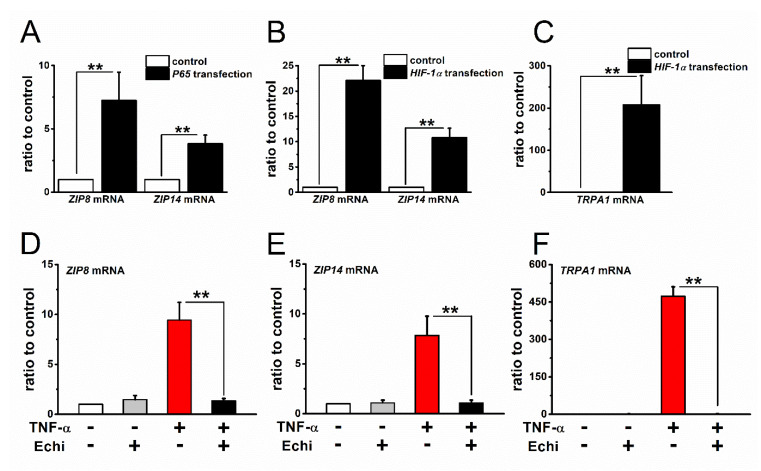
Expression of Zn^2+^ transporters in FLSs and involvement of transcriptional factors NF-κB and HIF-1α in inflammatory induction. (**A**) Expression of *ZIP8* and *ZIP14* mRNA in FLSs transfected with and without a P65 expression vector (mean ± SEM; four independent experiments; ** *p* < 0.01). (**B**) The expression of *ZIP8* and *ZIP14* mRNA in FLSs transfected with and without an HIF-1α expression vector (mean ± SEM; three independent experiments; ** *p* < 0.01). (**C**) As a control, the mRNA expression of *TRPA1* was also compared in FLSs with and without HIF-1α expression vector (mean ± SEM; three independent experiments; ** *p* < 0.01). Effects of Echi (1 μM) on the induction of (**D**) *ZIP8*, (**E**) *ZIP14*, and (**F**) *TRPA1* mRNA in FLSs stimulated with TNF-α. FLSs were treated with and without 10 U TNF-α for 24 h and the mRNA transcripts of ZIP8, ZIP14, and TRPA1 were compared. Echi treatment was performed for 24 h with and without TNF-α (mean ± SEM; four independent experiments; ** *p* < 0.01).

Among multiple cellular Zn^2+^ regulators, here we focused on plasma membrane-associated Zn^2+^ carriers that are involved in the observed basal [Zn^2+^]_i_ elevation in inflammatory FLSs. Among the 17 out of 24 known Zn^2+^ transporters, which are expressed in plasma membrane, exposure of FLSs to 100 U IL-1α and 10 U TNF-α for 24 h eventually increased the expression of only *ZIP8* and *ZIP14* mRNA transcripts (Supplementary Figure S2A–D). Because IL-1α and TNF-α can both activate NF-κB signaling cascades that are known to predominantly regulate transcriptional gene expression, we tested the involvement of NF-κB in the upregulation of these genes. To this end, we directly transfected FLSs with a *P65* plasmid vector that encodes RelA, an active component of NF-κB (Figure 6A). The transfected FLSs showed a clear increase in the expression of *ZIP8* and *ZIP14* mRNA. In addition, the inflammatory stimulation of FLSs is known to activate the transcriptional factor HIF-1α via NF-κB signaling in a transcriptional and/or post-transcriptional manner [26]. Therefore, we further examined the involvement of HIF-1α in the induction of *ZIP8* and *ZIP14* mRNA in FLSs. Transfection of FLSs with an *HIF-1α* plasmid vector effectively increased the expression of *ZIP8* and *ZIP14* mRNA, as well as *TRPA1* mRNA (Figure 6C), even without stimulation with TNF-α and IL-1α, as shown in Figure 6B. Moreover, when the FLSs were pretreated with the HIF inhibitor Echi (1 μM), the induction of *ZIP8* and *ZIP14* as well as *TRPA1* by TNF-α was abolished (Figure 6D–F), which suggested that NF-κB signaling and downstream activation of HIF-1α are critical for the inflammatory induction of ZIP8 and ZIP14. Taken together, these results, which are in overall agreement with our previous study on TRPA1 [26], indicate that HIF-1α is a novel transcriptional factor for ZIP8 and ZIP14. It is noteworthy that other Zn^2+^ regulators, such as organellar Zn^2+^ transporters and binding proteins, may be involved in inflammation-induced basal [Zn^2+^]_i_ elevation in FLSs.

### 2.5. Basal [Zn^2+^]_i_ Components in ZIP8- and ZIP14-Expressing Cells

**Figure 7:**
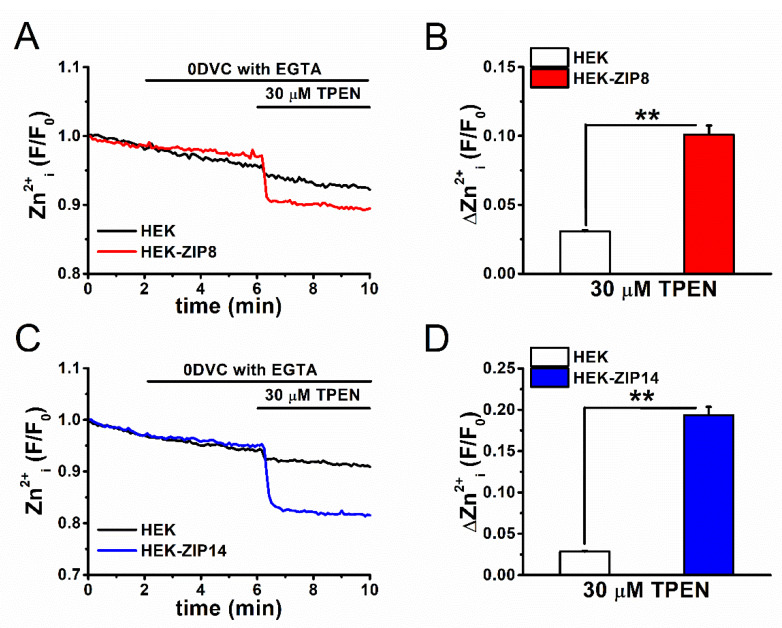
The basal Zn^2+^ components in ZIP8- and ZIP14-expressing cells. TPEN-sensitive basal Zn^2+^ components assayed with FluoZin-3 were compared between HEK cells with and without expression of (**A**,**B**) ZIP8 and (**C**,**D**) ZIP14. Extracellular Ca^2+^ and Zn^2+^ was removed by 0DVC with EGTA and cells were treated with 30 μM TPEN. (**B**) Fluorescence signal was measured as Zn^2+^_i_ (F/F_0_) and (**D**) the peak change by TPEN was plotted (mean ± SEM; B: *n* = 153 and *n* = 153 for HEK and HEK-ZIP8 cells, respectively, and three independent experiments each; ** *p* < 0.01; D: *n* = 153 and *n* = 153 for HEK and HEK-ZIP14 cells, respectively, and three independent experiments each; ** *p* < 0.01).

Finally, we confirmed that the heterologous expression of ZIP8 and ZIP14 in HEK cells elevates basal [Zn^2+^]_i_. Control, ZIP8-, and ZIP14-expressing HEK cells were treated with 30 μM TPEN after the removal of extracellular Ca^2+^ and Zn^2+^ with EGTA (0DVC with EGTA), and the [Zn^2+^]_i_ levels were monitored using FluoZin-3, as shown in Figure 7. TPEN, but not 0DVC, was found to induce relatively larger reduction in basal [Zn^2+^]_i_ components in HEK-ZIP8 (Figure 7A,C) and HEK-ZIP14 cells (Figure 7B,D) as compared to the control. This demonstrates that active rapid Zn^2+^ influx through ZIP8 and ZIP14 was minimal within this recording range due to the negligible reduction by 0DVC. However, [Zn^2+^]_i_ accumulation, followed by the overexpression of ZIP8 and ZIP14, was substantial in ZIP8- and ZIP14-expressing cells due to the significant reduction mediated by TPEN.

## 3. Discussion

In this study, we provide evidence that the transcription factor HIF-1α critically regulates the expression of ZIP8 and ZIP14 in inflammatory FLSs and propose that transcriptionally upregulated Zn^2+^ carriers ZIP8, ZIP14, and TRPA1 can change [Zn^2+^]_i_ levels in inflammation. Primary findings that are based on Fura-2/TPEN/Pyr assays identified a significant increase in the basal [Zn^2+^]_i_ concentration in TRPA1-expressing HEK cells and inflammatory FLSs. More importantly, it is a novel finding that TNF-α and IL-1αupregulate *ZIP8* and *ZIP14* expression via NF-κB signaling and downstream HIF-1α. This overexpression of ZIP8 and ZIP14 resulted in the accumulation of basal [Zn^2+^]_i_, which may act as an endogenous agonist and activate TRPA1. Taken together, our observations suggest that inflammation upregulates the transcription of ZIP8 and ZIP14, as well as TRPA1, in FLSs; and these Zn^2+^ carriers could change basal [Zn^2+^]_i_ in inflammatory cells.

In our previous study, we showed that Zn^2+^ is an endogenous TRPA1 agonist in inflammatory FLSs [26]. In addition, Zn^2+^ has been proposed to be an endogenous TRPA1 agonist when [Zn^2+^]_i_ concentration is higher than the nanomolar levels [27,29]. In fact, the EC_50_ of [Zn^2+^]_i_ that is required for the activation of TRPA1 in an inside-out patch configuration was reported to be 7.5 nM [27] and 50 nM [29], although the reason for this inconsistency is not clear. On the other hand, the present data show that inflammation significantly increased the basal [Zn^2+^]_i_ concentration from 0.09 to 0.48 nM in FLSs. Therefore, [Zn^2+^]_i_ that is close to the plasma membrane might show local increases in inflammatory FLSs and/or [Zn^2+^]_i_ may co-activate TRPA1 with other endogenous agonists. Indeed, the intrinsic Ca^2+^ oscillations in inflammatory FLSs were partially inhibited by treatment with catalase, an H_2_O_2_ scavenger [26]. Additionally, the oscillatory elevation of [Ca^2+^]_i_ in inflammatory FLSs could potentiate Zn^2+^-dependent activation of TRPA1, since [Ca^2+^]_i_ [30,31] positively modulates TRPA1 activity.

Active TRPA1 potentially carries Zn^2+^ into inflammatory FLSs. In our experiments, an AITC-induced increase in [Zn^2+^]_i_ was detected in FLSs with TRPA1, when 1 μM Zn^2+^ was added to the SBS (Figure 5D). On the other hand, the removal of external Zn^2+^ ([Zn^2+^]_o_) from the SBS reduced Zn^2+^ signals in TRPA1-expressing HEK cells (Figure 2D). These observations suggest that high expression levels of TRPA1 induce an inward flux of Zn^2+^, even when [Zn^2+^]_o_ in SBS is less than 0.15 μM (Supplementary Figure S1). This mode of active transport would result in a higher basal [Zn^2+^]_i_ concentration in TRPA1-expressing HEK cells (1.46 nM) when compared to control (0.03 nM). The treatment of mouse dorsal root ganglion (DRG) neurons with 30 and 300 μM Zn^2+^ has been shown to induce TRPA1-mediated Zn^2+^ influx [29]. The total zinc concentration in synovial fluid is reported to be ~1.7 and ~2.6 μM in healthy subjects and patients with rheumatoid arthritis, respectively [32]. However, the concentration of free Zn^2+^ might be lower than 1 μM, due to the presence of metal-binding proteins in the synovial fluid. Nevertheless, TRPA1 functions as an active carrier of Zn^2+^ in inflammatory FLSs, since this channel is intrinsically activated in these cells (Figure 1A). The TRPA1-dependent Ca^2+^ response has been reported to be more sensitive to [Zn^2+^]_o_ (>0.1 μM) in FLSs [26] when compared to that in mouse DRG (>5–30 μM) [27,29] and vagal pulmonary sensory (VPS) (>1 μM) [33] neurons. These reports collectively suggest that the oscillatory active TRPA1 channels act as carriers of small but sufficient amounts of Zn^2+^ for the facilitation of channel activity in FLSs, even when the [Zn^2+^]_o_ concentration is low (~0.15 μM, Supplementary Figure S1). In contrast, 1–10 μM [Zn^2+^]_o_ was sufficient for the facilitation in mouse DRG and VPS neurons, which was probably due to the low basal activity of TRPA1 in these cells. An alternative, and far more likely, scenario could be that human TRPA1 is more sensitive to [Zn^2+^]_i_ and/or [Zn^2+^]_o_ as compared to the murine analog of this channel (Matsubara and Muraki, unpublished data). It is notable that the control of [Zn^2+^]_i_ is complex and multiple regulators affect the cellular Zn^2+^ pool. The upregulation of TRPA1 and/or inflammation itself can modify cellular redox, which is sensitive to Zn^2+^ pool. In fact, the expression of metallothioneins changes the balance between the binding and unbinding of Zn^2+^. Because these factors have a large capacity for Zn^2+^ buffering, TRPA1, ZIP8, and ZIP14 may play a minor role as [Zn^2+^]_i_ determinants, even under inflammation. Further extensive analyses are required for an understanding of the physiological and pathophysiological roles of TRPA1, ZIP8, and ZIP14 as Zn^2+^ carriers,

In the present study, we estimated the basal [Zn^2+^]_i_ concentration to be 1.46 and 0.03 nM in HEK cells with and without TRPA1, respectively; and 0.48 and 0.09 nM in FLSs with (inflammatory) and without TRPA1 (no inflammation), respectively, utilizing Fura-2, TPEN, and Pyr. Fura-2 showed the highest affinity for Zn^2+^ among the tested synthetic Zn^2+^ indicators (K_d-Zn_: 2 nM) in spite of the disadvantages that are associated with sensitivity to a physiological concentration of Ca^2+^, and, therefore, it useful for the measurement of [Zn^2+^]_i_ in sub-nanomolar concentrations. Moreover, the ratiometric readouts that were obtained from the Fura-2 assay enabled us to compare [Zn^2+^]_i_ levels between different cells. The values that we estimated are in agreement with estimates in cells with physiological [Zn^2+^]_i_ concentration in the picomolar to nanomolar range, using FluoZin-3 and ZnAF-2, both of which are highly selective for Zn^2+^ [34,35]. Moreover, biosensors with high affinity and selectivity to Zn^2+^, which were developed by Vinkenborg et al. [28], have yielded basal [Zn^2+^]_i_ concentration estimates of 0.4 and 0.27 nM in pancreatic beta-cells and HEK cells, respectively. Therefore, the basal [Zn^2+^]_i_ concentrations that have been reported in the present study are in overall agreement to earlier reports, suggesting that Fura-2/TPEN/Pyr are useful for estimating and comparing basal [Zn^2+^]_i_ concentration values. However, the limitations of using Fura-2 were clear, since changes in [Ca^2+^]_i_ were observed to affect the signal. Therefore, the development of synthetic ratiometric indicators with high affinity and selectivity for Zn^2+^ is crucial for monitoring the dynamic changes in physiological [Zn^2+^]_i_ concentration.

Notably, the expression of ZIP8 and ZIP14 was found to be transcriptionally regulated by HIF-1α in FLSs under inflammatory conditions. In human lung epithelial cells, the overexpression of ZIP8 that is elicited by TNF-α has been shown to induce increase in [Zn^2+^]_i_, although the underlying mechanisms remain elusive [5]. On the other hand, the treatment of mouse hepatocytes with IL-1β caused an upregulation in ZIP14 expression via the induction of inducible nitric oxide synthase and downstream activation of activator protein-1 [36]. The upregulation in *ZIP8* and *ZIP14* expression in inflammatory FLSs on transfection with a *P65* expression vector, an active component of NF-κB, suggests the involvement of NF-κB signaling. Moreover, HIF-1α is critical for this regulation, as deduced from the observed upregulation in *ZIP8* and *ZIP14* on transfection with a HIF-1α expression vector and the attenuation of the former in the presence of the HIF inhibitor Echi. Inflammatory stimuli, such as TNF-α, IL-1β, and lipopolysaccharides, which mediate NF-κB signaling, are known to increase HIF-1α levels at the gene and/or protein level [37,38,39]. In FLSs, TNF-α and IL-1α are both known to increase the expression of HIF-1α, but not HIF-2α, at the protein level [26]. Therefore, HIF-1α could act as a transcription factor of *ZIP8* and *ZIP14*, and their overexpression that is induced by TNF-α and IL-1α results in an increase in basal [Zn^2+^]_i_ in FLSs. In the present study, we did not identify any potential binding sites of HIF-1α on the promoters of *ZIP8* and *ZIP14*. However, the transcription factor ATF4 potentially binds a region from -94 to -89 relative to the transcription start site (TSS) of the *ZIP14* promoter for upregulation [10]. Moreover, NF-κB is known to bind four consensus sites (κB1-κB4), which correspond to a 100 bp region proximal to the TSS of the *ZIP8* promoter, and the second binding site κB2 is critical for transcriptional activity [8]. NF-κB activated by TNF-α and IL-1α potentially interacts with κB2 and upregulates ZIP8 expression because κB2 is included in the promoter region of human *ZIP8* in FLSs. Further investigation is required to elucidate the interaction between NF-κB and HIF-1α that underlies the upregulation of ZIP8 in inflammatory FLSs.

In the previous study, we showed that the activation of TRPA1 by AITC reduced the inflammatory production of IL-6 and IL-8 in FLSs [26]. In addition, the inhibition of basal activity of TRPA1 increased IL-8 production. Therefore, the activation of TRPA1 by Zn^2+^, which is transported by TRPA1 and ZIPs, may contribute to reducing the inflammatory response in FLSs. Coordinate transport of Zn^2+^ through TRPA1 and ZIPs is a potential feedback mechanism against inflammatory conditions via the activation of TRPA1 because it is well known that TRPA1 activation causes inflammatory pain. In addition, 0VDC with EGTA effectively reduced the basal Zn^2+^ in HEK cells with TRPA1 (Figure 2C,D), but not with ZIP8 and ZIP14 (Figure 7). It is possible that Zn^2+^ via TRPA1 is involved in Zn^2+^-dependent cellular processes within minutes and/or an hour. Nevertheless, the contribution of ZIP8 and ZIP14 as well as TRPA1 to the processes may be exclusive and redox signaling and Zn^2+^ buffering play dominant roles in cellular Zn^2+^ regulation [40].

In conclusion, inflammatory conditions transcriptionally upregulate ZIP8 and ZIP14, as well as TRPA1 expression in FLSs via NF-κB signaling and the downstream activation of HIF-1α. Therefore, these Zn^2+^ carriers could regulate basal [Zn^2+^]_i_ levels in inflammatory FLSs.

## 4. Experimental Procedures

### 4.1. Reagents

The following drugs were used: allyl isothiocyanate (AITC; Kanto Chemical Co., Tokyo, Japan), ZnSO_4_ (Zn^2+^; Wako Pure Chemical Co., Osaka, Japan), HC-030031 (HC; Enzo Life Sciences, Farmingdale, NY, USA), A-967079 (A96; Sigma-Aldrich, Tokyo, Japan), zinc-pyrichione (Pyr; Sigma/Aldrich), echinomycin (Echi; Sigma/Aldrich), TNF-α (Wako Pure Chemical Co.), IL-1α (Wako Pure Chemical Co.), and N,N ‘,N’,N-tetrakis (2-pyridylmethyl) ethylenediamine (TPEN; Wako Pure Chemical Co.). Each drug was dissolved in the vehicle that was recommended by the manufacturer.

### 4.2. Cell Culture

Human FLSs were purchased from Cell Applications and cultured in Synoviocyte Growth Medium containing 10% growth supplement, 100 U/mL penicillin G (Meiji Seika Pharma Co., Ltd., Tokyo, Japan), and 100 μg/mL streptomycin (Meiji Seika Pharma Co., Ltd.), as described previously [26]. The cultured cells were maintained at 37 °C in a 5% CO_2_ atmosphere. After FLSs acquired 70–80% confluence, the cells were reseeded once every 10 days until nine passages were complete. The cells that grew with a doubling time of 6–8 days after this stage were comprised of a homogenous population, in which induction of TRPA1 by cytokine treatment was found to be unaffected. For experiments, the reseeded cells were cultured for 16 days and then exposed to TNF-α and IL-1α. Human embryonic kidney 293 (HEK) cell lines were obtained from the Health Science Research Resources Bank (HSRRB) and maintained in Dulbecco’s Modified Essential Medium (D-MEM, Sigma/Aldrich) that was supplemented with 10% heat-inactivated fetal calf serum (FCS, SAFC Biosciences Inc, Tokyo, Japan), penicillin G (100 U/mL), and streptomycin (100 μg/mL).

### 4.3. Quantitative PCR

Real-time quantitative PCR was performed using SYBR Green on a Thermal Cycler Dice Real Time System (Takara Bio Inc., Kusatsu, Japan), as described previously [26]. The transcriptional quantification of gene products was carried out by normalization to that of *β-ACTIN*. Each cDNA sample was tested in duplicate or triplicate. The program that was used for quantitative PCR amplification included a 30 s activation of Ex Taq™ DNA polymerase at 95 °C, a 15 s denaturation step at 95 °C, a 60 s annealing and extension step at 60 °C (for 45 cycles), and a dissociation step (15 s at 95 °C, 30 s at 60 °C, and 15 s at 95 °C). Supplementary Table S1 shows the oligonucleotide sequences of primers specific for human *TRPA1*, *ZNT1-8*, *ZIP1-14*, and *β-ACTIN*.

### 4.4. Recombinant Expression of Wild and Mutant TRPA1, P65, HIF-1α, ZIP8, and ZIP14 in HEK Cells and FLSs

The partially confluent HEK cells were transfected with pcDNA3.1/neo(+) (pcDNA3.1, for [Ca^2+^]_i_ and [Zn^2+^]_i_ measurements) and pIRES2-AcGFP1 (for patch-clamp experiments) plasmids containing human wild and mutant TRPA1, pcDNA3.1-human ZIP8 plasmid DNA, and pcDNA3.1-human ZIP14 plasmid DNA using Lipofectamine 3000 (Thermo Fisher Scientific, Yokohama, Japan). The FLSs were similarly transfected with pCMV-human P65 plasmid DNA and pcDNA3.1/hyg(+)-human HIF-1α plasmid DNA using Lipofectamine 3000. The TRPA1 mutation was constructed by PCR using mutant oligonucleotide primers, in which an aspartic acid residue at 915 was changed to alanine (Agilent Technologies, Santa Clara, CA, USA). All of the experiments were performed within 48 h of transfection. Sequencing verified the construct.

### 4.5. Patch Clamp Electrophysiology

Whole-cell current recordings were performed, as described previously [41]. The resistance of the electrodes was 3–5 MΩ when filled with pipette solution. A Cs^+^-rich pipette solution contained (in mM) Cs-aspartate 110, CsCl 30, MgCl_2_ 1, HEPES 10, EGTA 10, and Na_2_ATP 2 (adjusted to pH 7.2 with CsOH). The [Ca^2+^]_i_ concentration was adjusted to a pCa value of 6.5 (0.3 μM Ca^2+^) by adding CaCl_2_ to the pipette solution to maintain TRPA1 currents. Membrane currents and voltage signals were digitized using an analogue-digital converter (PCI-6229, National Instruments Japan, Tokyo, Japan). The WinWCPV4.5 software was used for data acquisition and the analysis of whole-cell currents (developed by Dr. John Dempster, University of Strathclyde, UK). The liquid junction potential between the pipette and bath solutions (−10 mV) was corrected. A ramp voltage protocol from −110 to +90 mV for 100 ms was applied every 5 s from a holding potential of −10 mV. The leak current component was not subtracted from the recorded currents. A standard HEPES-buffered bathing solution (SBS (in mM): NaCl 137, KCl 5.9, CaCl_2_ 2.2, MgCl_2_ 1.2, glucose 14, HEPES 10 (adjusted to pH 7.4 with NaOH)) was used. All of the experiments were performed at 25 ± 1 °C.

### 4.6. Measurement of Ca^2+^ Fluorescence Ratio and Auto-Fluorescence Images of Cells

The changes in [Ca^2+^]_i_ concentrations were monitored with Fura-2, as described previously [41]. The cells were loaded with 10 μM Fura-2-acetoxymethyl ester (Fura2-AM; Dojindo Molecular Technologies, Inc, Kumamoto, Japan) in SBS for 30 min. at room temperature. Fura-2 fluorescence signals were measured at 0.2 Hz in an ARGUS/HiSCA imaging system (Hamamatsu Photonics, Hamamatsu, Japan) that was operated using the Imaging Workbench software v6.0 (INDEC Medical Systems, Santa Clara, CA), and the fluorescence ratio (Ca^2+^_i_ (F_340_/F_380_)) was calculated. For each analysis, the whole cell area was chosen as the region of interest to obtain the averaged values of fluorescence ratio. In the present study, we used a zero divalent cation (0DVC) bathing solution where both CaCl_2_ and MgCl_2_ were removed from SBS in the presence (Figure 4 and Figure 7) or absence (Figure 2 and Figure 3) of 1 mM EGTA. All of the experiments were performed at 25 ± 1 °C. For the recording of auto-fluorescence images, the cells were excited with light of 488 nm wavelength and the emitted fluorescence was collected at wavelengths longer than 505 nm in a confocal laser scanning microscope (LSM 510 META, Carl Zeiss, Oberkochen, Germany).

### 4.7. Fluorescence-Based Measurement of Zn^2+^

The cells were incubated with 10 μM Fura-2-AM in SBS for 30 min. at room temperature. During the estimation of [Zn^2+^]_i_ using Fura-2 (Figure 4), 1 mM EGTA was added to remove residual Ca^2+^ and Zn^2+^ in 0DVC. Fura-2 fluorescence signals were measured at 0.2 Hz using the Argus/HisCa imaging system that was driven by Imaging Workbench v6.0, and the corresponding fluorescence ratio (Zn^2+^_i_ (F_340_/F_380_)) was calculated. In order to generate a mathematical relationship between the fluorescence intensity ratio and Zn^2+^ concentration, cells that were permeabilized with the Zn^2+^ ionophore Pyr were incubated with Zn^2+^ at a concentration range of 1–300 nM using the Zn^2+^-EGTA buffer. The change in ratio (ΔZn^2+^_i_ (F_340_/F_380_)) normalized to that in the presence of TPEN was plotted against the corresponding Zn^2+^ concentration, and the generated data set was fitted to the following equation using the Origin J9.1 software (LightStone, Tokyo, Japan):

ΔR = ΔR_max_*Zn^2+^concentration/[K_d_+Zn^2+^concentration]

where ΔR is ΔZn^2+^_i_ (F_340_/F_380_); and, K_d_ and ΔR_max_ are the dissociation constants of Fura-2 in the presence of Zn^2+^ and maximal ΔZn^2+^_i_ (F_340_/F_380_), respectively. The change in [Zn^2+^]_i_ was also measured using FluoZin-3. The cells were incubated with 10 μM FluoZin-3AM in SBS for 30 min. at room temperature. When the cells were excited with light of 488 nm wavelength, FluorZin-3 fluorescence signals that were emitted at wavelengths longer than 510 nm were collected at 0.2 Hz using the Argus/HisCa imaging system driven by Imaging Workbench v6.0, and the changes in the fluorescence intensity were calculated at time zero (Zn^2+^_i_ (F/F_0_)). For each analysis, the whole cell area was chosen as the region of interest for averaging the fluorescence signals. For the quantitative measurement of changes in Zn^2+^ levels, we collected each cell on a single coverslip for analysis of HEK cells and FLSs (the total numbers (*n*) shown in figure legends), and then repeated the same experiment with other coverslips to reduce variation (the total number of independent experiments shown in figure legends).

### 4.8. Estimation of Zn^2+^ Concentration in SBS

The Zn^2+^ concentration in SBS was measured using a Metallo Assay Kit (Metallogenics Co., Ltd., Chiba, Japan), while following the manufacturer’ s protocol. The absorbance at 570 nm was determined to be between 0.153 and 3 μM Zn^2+^, and an absorbance-Zn^2+^ concentration relationship was constructed to confirm the concentration of Zn^2+^ in SBS.

### 4.9. Statistical Analysis

Origin J9.1 was used for data analysis and representation. The data reported are expressed as the mean ± SEM, where *n* indicates the total number of cells in independent experiments. The statistical significance between two groups and among multiple groups was examined using paired or unpaired Student’s *t*-test and one-way ANOVA or Tukey-Kramer test, respectively. For all of the tests, the *p*-values below 0.05 were considered to be statistically significant.

## Data Availability

Not applicable.

## Supplementary Materials

Supplementary Materials are available online at https://www.mdpi.com/article/10.3390/ijms22126349/s1.

## Abbreviations

[Zn^2+^]_i_	intracellular free zinc
HIF-1α	hypoxia-induced factor 1α
TRPA1	transient receptor potential ankyrin 1
FLSs	human fibroblast-like synoviocytes
TNF-α	tumor necrosis factor-α
IL-1α	interleukin-1α
NF-κB	nuclear factor-κB
[Zn^2+^]_e_	extracellular free Zn^2+^
