# Supplementary material for: HIF-1α Dependent Upregulation of ZIP8, ZIP14, and TRPA1 Modify Intracellular Zn2+ Accumulation in Inflammatory Synoviocytes
Source: Int J Mol Sci. 2021 Jun 14;22(12):6349. doi: 10.3390/ijms22126349 (PMC8231863; doi:10.3390/ijms22126349)
Supplement: Supplementary file 1 [file ijms-22-06349-s001.zip › ijms-1211401-supplementary.pdf]

Supplementary Information

HIF-1α dependent upregulation of ZIP8, ZIP14, and TRPA1 modify intracellular Zn^{2+} accumulation in inflammatory synoviocytes

Noriyuki Hatano[1], Masaki Matsubara[1], Hiroka Suzuki[1], Yukiko Muraki[1], Katsuhiko Muraki[1*]

[1]Laboratory of Cellular Pharmacology, School of Pharmacy, Aichi-Gakuin University, 1-100 Kusumoto, Chikusa, Nagoya 464-8650, Japan

*Corresponding author: Prof. Katsuhiko Muraki
E-mail: kmuraki@dpc.agu.ac.jp

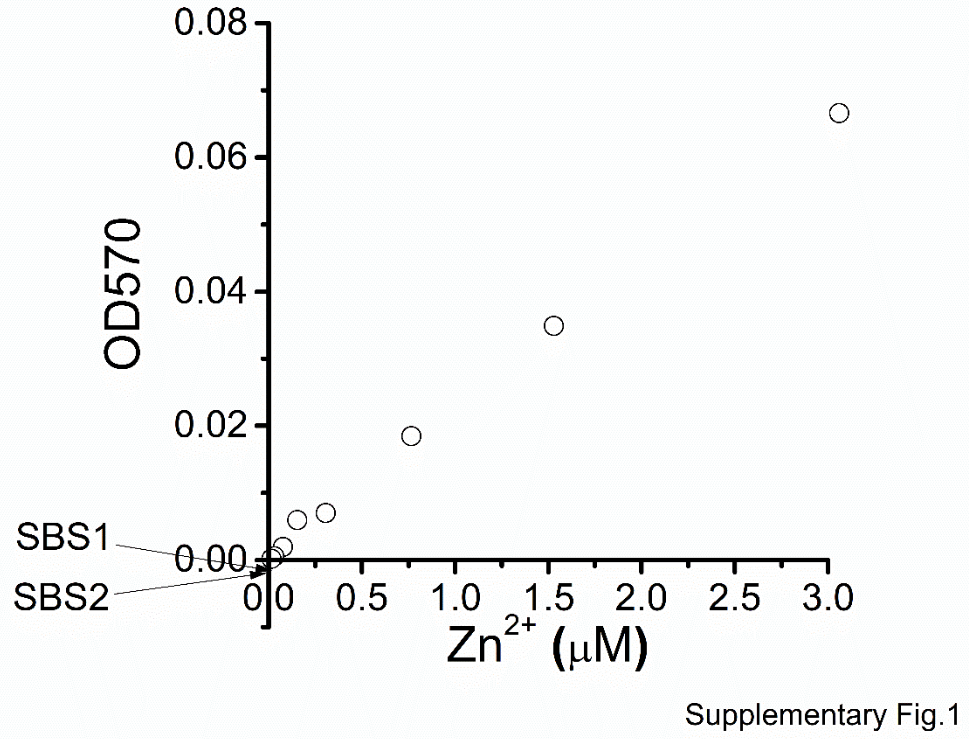

Supplementary Figure S1. Zn^{2+} concentration in the SBS. Zn^{2+} concentration was measured using a Metalloassay Kit. The absorbance at 570 nm was determined to be within 0.153 for 3 μM Zn^{2+} and this was used to determine the relationship between absorbance and Zn^{2+} concentration. Two independent SBSs were assayed, and the absorbances of SBS1 and SBS2 were -0.00115 and -0.00165, respectively.

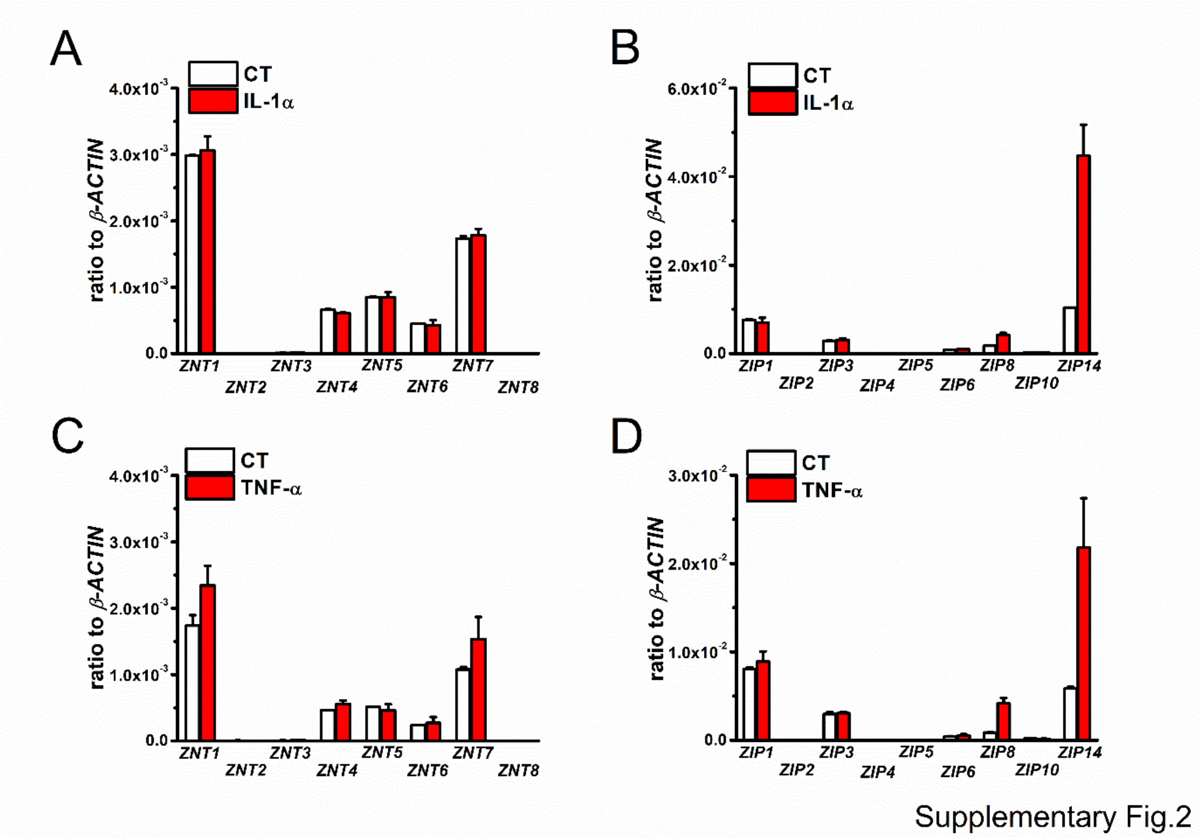

Supplementary Figure S2. Expression of membrane-associated Zn^{2+} transporters in FLSs with and without inflammation. FLSs were treated with and without **(A, B)** 100 U IL-1α or **(C, D)** 10 U TNF-α for 24 h. The mRNA transcripts of 17 out of 24 known Zn^{2+} transporters were compared between control (CT, 2 independent experiments) and inflammatory FLSs (IL-1α and TNF-α, 2 independent experiments).

Supplementary Table S1. Primer sets for *TRPA1*, *ZNT1-8*, *ZIP1-14*, and *β-ACTIN*

gene	GenBank accession No.	amplicon		
TRPA1	NM_007332	140 bp	sense	TGCATGTTGCATTCCACAGAAG
			antisense	TTGAGGGCTGTAAGCGGTTCATA
ZIP1	NM_014437	111 bp	sense	CTCAGCTTCCCGCCAGAA
			antisense	TCATCTATGGCAGCCAGGTAGTC
ZIP2	NM_014579	101 bp	sense	TTTGGAGTCGCTGGCATTG
			antisense	GGCTGTGGAGTTCGAAGATATGA
ZIP3	NM_144564	101 bp	sense	CGTGAAGATCATCGAGACAGATTT
			antisense	AGCACGTGGCCAGAAACAC
ZIP4	NM_017767	113 bp	sense	CCCTGTGCCCCTCATCAG
			antisense	GGGTCACCCCAGCCTGTT
ZIP5	NM_173596	121 bp	sense	AGGAATCTCGAAACACGCAACT
			antisense	GTGGGTGCTGGCTGTTCTTC
ZIP6	NM_012319	111 bp	sense	TGGAATGGCAACAGGAATTTT
			antisense	GGTACCATATCAACCAGAGCAACA
ZIP8	NM_022154	111 bp	sense	CAAATCCTGCTGTCACAGAAGCT
			antisense	GCCCCTTCAAACAGGTACATG
ZIP10	NM_001127257	121 bp	sense	GTCATTAGCCTGCTTTCCTTGCT
			antisense	GGGCGTCTCCACTCATTGTT
ZIP14	NM_001128431	111 bp	sense	CAACTGGATTTTTGCGCTAGCT
			antisense	AAGATGCTGCCCTTCCTTTCA
ZNT1	NM_021194	121 bp	sense	TTTCTGCATGTCCTTGGAGATG
			antisense	CAGGGAAACATGGATTCACACA
ZNT2	NM_001004434	101 bp	sense	CTGTCTGTCCACATCGCCATT
			antisense	GTCACGGTGTGGAAGTGGAACT
ZNT3	NM_003459	123 bp	sense	CCTCATCTACTTCAAGCCTCAATACA
			antisense	CCTTCCATGAGGATTCGAAGAA
ZNT4	NM_013309	131 bp	sense	GACCATTGCTGCCGTTCTGT
			antisense	GGGTGAGTATGATGGCGCTTA
ZNT5	NM_022902	121 bp	sense	GGCCTGATCTCGGATGGATT
			antisense	TTCGGCCGTACCCATAGGA
ZNT6	NM_001193513	111 bp	sense	TCAACCTGTTCACGATGCTTTC
			antisense	ACAAGCTTCGACTAAGATCTGCAA
ZNT7	NM_133496	101 bp	sense	TGTTTTTCTTCCTGTGCCTGAA
			antisense	CATGTGAAAAGAGTCGGAAATCAA
ZNT8	NM_173851	131 bp	sense	CTCCAAGCCCACAGAAAAGG
			antisense	CAGCAAGACTCCCAGCAATG
β-ACTIN	NM_001101	112 bp	sense	ACCGAGCGCGGCTACA
			antisense	CAGCCGTGGCCATCTCTT

supplementary Table1
